# Informing efficient randomised controlled trials: exploration of challenges in developing progression criteria for internal pilot studies

**DOI:** 10.1136/bmjopen-2016-013537

**Published:** 2017-02-17

**Authors:** Kerry N L Avery, Paula R Williamson, Carrol Gamble, Elaine O'Connell Francischetto, Chris Metcalfe, Peter Davidson, Hywel Williams, Jane M Blazeby

**Affiliations:** 1 Medical Research Council ConDuCT-II Hub for Trials Methodology Research, School of Social and Community Medicine, University of Bristol, Bristol, UK; 2 Medical Research Council North West Hub for Trials Methodology Research, Department of Biostatistics, University of Liverpool, Liverpool, UK; 3 National Institute for Health Research Evaluation, Trials and Studies Coordinating Centre, University of Southampton, Southampton, UK; 4 Centre of Evidence-Based Dermatology, University of Nottingham, Queen's Medical Centre, Nottingham University Hospitals NHS Trust, Nottingham, UK; 5 Division of Surgery, Head and Neck, University Hospitals Bristol NHS Foundation Trust, Bristol, UK

**Keywords:** STATISTICS & RESEARCH METHODS

## Abstract

**Objectives:**

Designing studies with an internal pilot phase may optimise the use of pilot work to inform more efficient randomised controlled trials (RCTs). Careful selection of preagreed decision or ‘progression’ criteria at the juncture between the internal pilot and main trial phases provides a valuable opportunity to evaluate the likely success of the main trial and optimise its design or, if necessary, to make the decision not to proceed with the main trial. Guidance on the appropriate selection and application of progression criteria is, however, lacking. This paper outlines the key issues to consider in the optimal development and review of operational progression criteria for RCTs with an internal pilot phase.

**Design:**

A structured literature review and exploration of stakeholders' opinions at a Medical Research Council (MRC) Hubs for Trials Methodology Research workshop. Key stakeholders included triallists, methodologists, statisticians and funders.

**Results:**

There is considerable variation in the use of progression criteria for RCTs with an internal pilot phase, although 3 common issues predominate: trial recruitment, protocol adherence and outcome data. Detailed and systematic reporting around the decision-making process for stopping, amending or proceeding to a main trial is uncommon, which may hamper understanding in the research community about the appropriate and optimal use of RCTs with an internal pilot phase. 10 top tips for the development, use and reporting of progression criteria for internal pilot studies are presented.

**Conclusions:**

Systematic and transparent reporting of the design, results and evaluation of internal pilot trials in the literature should be encouraged in order to facilitate understanding in the research community and to inform future trials.

**Strengths and limitations of this study**

- To the best of our knowledge, this is the first study to explore and summarise the key issues to consider when developing and using progression criteria for randomised controlled trials with an internal pilot phase, where guidance was previously lacking.
- This study was informed by a structured search of the trials literature and explored the opinions of a large number and wide variety of key stakeholders, including triallists, methodologists, statisticians and funding bodies.
- The work was undertaken in the UK and is illustrated with examples from a number of National Institute for Health Research (NIHR)-funded studies, which may not represent the full variability of trials observed in other contexts or settings.

## Introduction

Well-designed and conducted randomised controlled trials (RCTs) that are reported in full have the potential to change clinical practice and influence health policy and decision-making.^1^ Many trials, however, face challenges in design and conduct, for example, in relation to recruitment, adherence, outcome assessment, sample size and follow-up. Methods to improve the quality of trial conduct and the efficient use of resources by earlier identification of studies that cannot be successfully delivered are, therefore, important and may include the use of feasibility and pilot studies.^2–6^ A recent publication by Kistin and Silverstein^6^ has concisely outlined the many potential uses of pilot work, with emphasis on enhancing the efficiency and internal validity of the subsequent definitive trial. These include piloting key study logistics such as recruitment, intervention delivery and adherence and identifying potential barriers and facilitators to eventual intervention dissemination and implementation. While there are no universally accepted definitions of the different types of feasibility and pilot studies,^7–12^ a new conceptual framework has recently been proposed by Eldridge and colleagues for defining pilot and feasibility studies conducted in preparation for an RCT. This framework describes pilot studies as a subset of feasibility studies: a feasibility study asks ‘whether something can be done, should we proceed with it, and if so, how’,^13^ whereas a pilot study ‘may ask the same questions but also has a specific design feature: in a pilot study, a future study, or part of a future study, is conducted on a smaller scale’. This definition of a pilot study is similar in some ways to the definition proposed by the UK National Institute for Health Research, which describes how pilot studies may still have some feasibility objectives but focus largely on uncertainties around the processes of the main study; for example, to ensure that recruitment, randomisation, intervention and follow-up assessments all run smoothly,^12^ thereby allowing the methods used in the main study to be optimised. Using either set of definitions, two types of pilot study can be distinguished. An ‘external pilot’ is a rehearsal of the main study where the outcome data are not included as part of the main trial outcome data set. Alternatively, the pilot phase may form the first part of the trial and the outcome data generated may contribute to the final analyses. This is called an ‘internal pilot’ and is intended to offer advantages in terms of preserving efficiency in RCTs, preventing waste of valuable resources and avoiding recruiting participants to a trial that may not succeed.

A key milestone in a trial with an internal pilot phase is the progression from the pilot (first) phase to the main (second) trial phase. This juncture is important because it provides an opportunity to reflect on the viability of the trial by allowing RCT processes to be formally reviewed and decisions to be made about whether it is appropriate for the study to proceed to the main trial phase or whether it is necessary or possible to modify the operational aspects of the design to promote success. Ideally before the study starts, a trial team together with the funding body would identify prespecified ‘progression’ or ‘decision’ criteria that are used to indicate whether targets have been met during the internal pilot phase and the main trial should proceed.^12^ Rather than progression criteria being considered on a simple stop/go basis, it may be preferable to explore whether the trial can proceed with modifications, similar to a red/amber/green traffic light system: stop/red (eg, when there are intractable issues that cannot be remedied), amend/amber (where there are remediable issues, thereafter proceeding with caution) or continue/green (where there are no concerning issues that threaten the success of the trial). These prespecified criteria are set in order to judge the viability of completing the main trial within the planned timetable and budget and usually address the key areas of uncertainty or risk that could influence the success of the trial.

Establishing the progression criteria at the outset of the trial may be considered essential but there is little published advice about how to do this optimally. To address this uncertainty, the Medical Research Council (MRC) Hubs for Trials Methodology Research hosted a 1-day workshop to consider key issues in the development and review of progression criteria when the design of an RCT includes an internal pilot phase.

### Workshop methodology

Discussion at this workshop was informed by literature reviews undertaken by two MRC Hubs for Trials Methodology Research (ConDuCT-II and the North West), details of which have been published previously.^7^ First, PubMed was searched for articles with ‘pilot studies’ in the title (inception to 03/04/2013) to identify review and methodology papers published in English where pilot work was discussed. Of the 289 publications identified in the search, full-text articles of 40 papers were identified and definitions of pilot work were extracted and examined (KNLA, JMB, EOF). All terms used to describe pilot work and definitions were recorded verbatim. The terms identified in this initial review informed search strategies for searches of the top 10 impact factor medical journals that endorse CONSORT guidelines (*New England Journal of Medicine*; *The Lancet*; *Journal of the American Medical Association*; *Lancet Oncology*; *Journal of Clinical Oncology*; *Annals of Internal Medicine*; *PLOS Medicine*; *Circulation*; *The BMJ*; *Journal of the National Cancer Institute*) and the journal *Trials* between January 2011 and August 2013 (restricted to studies conducted in adults and published in English). Additionally, experts were contacted to identify studies that had not been published at the time of the search. The review aimed to identify different types of pilot work undertaken and how they might inform the design and progression to a main trial. Search terms included: Adaptive seamless; Adaptive trial; Early phase; External pilot; Development phase; Feasibility phase; Feasibility project; Feasibility study; Feasibility trial; In-built pilot; Internal pilot; Lead in study; Phase II trial; Pilot study; Pilot trial; Pilot phase; Pilot project; Pilot work; Pre-testing; Proof of concept; Proof of principle; Run in phase; Sample size re-estimation design; Vanguard trial. Publications reporting on full RCTs without pilot work, reviews, guidance documents and purely methodological studies were excluded. This review was considered structured rather than systematic, as it was not intended to summarise all available evidence on the subject but rather to collate a broadly representative set of papers reporting on pilot work to identify key issues to consider in the next phase of the study (described below).

After duplications were removed and experts were contacted for further details where necessary, a total of 309 publications were reviewed and 11 RCTs with an internal pilot phase were identified (table 1). Key findings were drawn from this review, which showed that it was difficult to identify trials with an internal pilot, perhaps due to poor reporting, lack of prioritisation by journals as a means to manage demand or because such trials were relatively uncommon until recently. Furthermore, reporting of the development and use of progression criteria in the identified internal pilot trials was often missing or lacking detail. These findings informed key discussion points at the workshop, including definitions identified in the first review, purpose and examples of RCTs with an internal pilot phase and points to consider in developing progression criteria from an internal pilot to a main trial. Discussions were minuted and summarised in a report for discussion within the study group (KNLA, JMB, EOF, PRW, CG). A detailed summary document was then circulated to all workshop attendees for comment and feedback.

**Table 1:** Examples of the variety of progression criteria described in RCTs with an internal pilot phase

		Progression criteria			
Name of RCT, year	Trial objective	Recruitment	Protocol adherence	Outcome data	Other
KORAL,^14^ 2010	Multicentre RCT to evaluate the effectiveness and cost-effectiveness of arthroscopic lavage for osteoarthritis of the knee	Proportion of eligible patients randomised, reasons for refusals	Not applicable/not specified	Not applicable/not specified	Not applicable/ not specified
SUN(^_^)D,^15^ 2012	Multicentre RCT to evaluate the strategic use of new generation antidepressants for depression	Demographic and clinical characteristics of screened and enrolled patients Randomisation success	Whether interventions as stipulated in the protocol are adhered to Whether blinding had been successfully implemented	Whether assessments are made with satisfactory follow-up rates Inter-rater and intra-rater reliability of primary outcomes	Safety aspects
EUROTHERM-3235,^16^ 2013	Multicentre RCT to evaluate therapeutic hypothermia for traumatic brain injury	Feasibility of recruitment (sites and patients) Patient eligibility—previous observational studies had predicted that 50% of patients would be eligible	Feasibility of the protocol, in particular the effectiveness of delivery of the cooling protocol	Not applicable/not specified	Not applicable/ not specified
TasP,^17^ 2013	Cluster-randomised community-based RCT to evaluate the impact of immediate vs WHO recommendations-guided antiretroviral therapy initiation on HIV incidence	Evidence that adequate statistical power for the trial will be achieved based on a number of parameters, including: baseline HIV prevalence; HIV incidence	Evidence that adequate statistical power will be achieved based on various parameters, including: initial and repeated HIV testing uptake; antiretroviral therapy uptake; proportion of participants who know their HIV status and who disclose their HIV status; migration between and out of clusters	Not applicable/not specified	Evidence that adequate statistical power for the trial will be achieved based on a number of parameters, including extent of sexual partnerships with people outside trial setting

		Progression criteria			
Name of RCT, year	Trial objective	Recruitment	Protocol adherence	Completeness and quality of outcome data	Other
By-Band-Sleeve,^18^ 2014	Multicentre RCT to compare the effectiveness and cost-effectiveness of Roux-en-Y gastric bypass and adjustable gastric band surgery to treat severe and complex obesity	60% of patients referred for bariatric surgery to be eligible for the trial (if necessary, revising the eligibility criteria) Initially, 30% and then 50% of patients consent to randomisation	<5% failure to receive allocated intervention	<15% lost to follow-up	Not applicable/ not specified
COMMANDO,^19^ 2014	Multicentre RCT to treat childhood obesity in the community using Mandolean therapy (a portable food weighing scale that provides feedback to participants to promote normal patterns of eating and satiety)	Recruitment of ≥36 families who would be eligible for the main study	90% of patients randomised to Mandolean therapy would be successfully eating off the device at least five times a week	≥80% would attend the 3-month nurse appointment for both study groups	≥60% of those using Mandolean therapy would demonstrate a decrease in speed of meal consumption (longer meals) since baseline within 3 months of starting therapy
NERVES,^20^ 2014	Multicentre RCT to evaluate the effectiveness and cost-effectiveness of transforaminal epidural steroid injection to surgical microdisectomy for acute sciatica	Recruitment of ≥30 patients in 6 months Consent rate of ≥40%	≤20% failure to receive the alternative intervention	<50% of patients in injection group proceed to surgery	Not applicable/ not specified
PLINY,^21^ 2014	Multicentre RCT to evaluate the effectiveness and cost-effectiveness of telephone befriending to maintain quality of life in older people	Recruitment of ≥68 participants in 95 days	Service provider (a national charity) should recruit, train and retain enough volunteers to deliver the telephone befriending service	Collect valid primary outcome data for 56 (80%) of those recruited at the 6 month follow-up	Not applicable/ not specified
Sheffield physical activity ‘Booster’,^22^ 2014	Single-centre community-based RCT to evaluate the effectiveness and cost-effectiveness of ‘booster’ interventions to sustain increased physical activity in middle-aged adults in 55 deprived urban neighbourhoods	60 participants recruited	≥70% of those randomised to Booster interventions receive interventions per protocol	≥75% of those recruited to provide 3 months follow-up measures, including primary outcome measure	Not applicable/ not specified
TARVA,^23^ 2014	Multi-centre RCT to compare the clinical and cost-effectiveness of total ankle replacement vs ankle arthrodesis	33 patients (10% of total target recruitment randomised in 6 months) 82 patients (25% of total sample size) recruited in 15 months Patient willingness to be randomised Surgeon willingness to randomise Whether requiring surgical competence in both procedures limits recruitment	Not applicable/ not specified	Not applicable/ not specified	Not applicable/ not specified
VIOLET,^24^ 2015	Multicentre RCT to compare video-assisted thoracoscopic lobectomy vs conventional open lobectomy for lung cancer	20 eligible patients screened per month 30% of eligible patients recruited initially, increasing to 50% within 6 months at each centre	Not applicable/ not specified	Not applicable/ not specified	Not applicable/ not specified

‘Not applicable/not specified’: no formal progression criteria proposed or detailed in trial report(s).

RCT, randomised controlled trial.

This paper summarises the main challenges when developing progression criteria for a trial with an internal pilot phase, illustrated with examples from a number of National Institute for Health Research (NIHR)-funded studies, to inform the optimal design of efficient RCTs.

### Predominant types of progression criteria in an RCT with an internal pilot phase

A variety of operational progression criteria may be used to assess the likely success of a trial with an internal pilot. However, three common issues predominate: trial recruitment, protocol adherence and outcome data (table 1). The first section of this paper considers each of these in turn, and approaches to the development and review of progression criteria, considering also the role of the Trial Steering Committee (TSC). Next, there are sections regarding opportunities for using changing evidence and incorporating this at the juncture of an internal pilot and main trial. This will allow the formal determination of optimal design and progression criteria for RCTs with an internal pilot phase. The reporting of internal pilot studies is also considered. Finally, 10 top tips for using progression criteria for internal pilot studies are presented.

### Progression criteria

#### Trial recruitment

Recruitment is most often the focus of progression criteria^14–16^ ^20–29^ and a concern for triallists and funders.^30^ In the internal pilot phase, investigators may, for example, estimate the expected prevalence or rate of incident cases in the population, the number expected to be screened for eligibility,^16^ ^25^ the number screened who are expected to be eligible^16^ ^25^ or the number eligible who are expected to consent to randomisation^14^ ^15^ ^25^ and have prespecified progression criteria related to these estimates. A common error when setting recruitment targets is to define a target number to be randomised by a specified time point. Trials frequently suffer from delays in the contracting and setting-up of sites that results in departure from the initial recruitment time plan. It may be more desirable to suggest a recruitment rate per centre per month.^31^ The recruitment rate for the pilot phase may thus be informed by the trial's recruitment trajectory for achieving the final sample size but recruitment to the substantive phase of the study may be remodelled later as part of the analysis of the internal pilot data.

The NIHR By-Band-Sleeve study is an example of a trial with an internal pilot phase that successfully progressed to a main trial.^18^ It was designed to compare the effectiveness and cost-effectiveness of Roux-en-Y gastric bypass, adjustable gastric band surgery and sleeve gastrectomy for severe and complex obesity. Preagreed recruitment-related progression criteria specified that: (1) 60% of patients referred for bariatric surgery would meet the eligibility criteria,^18^ and (2) initially, 30% of patients would consent to randomisation rising to 50% after 18 months as recruiters gained in experience and training with integration of a qualitative recruitment intervention. The internal pilot found that more patients than expected (74%) were eligible but that fewer than expected were randomised. The internal pilot data were used to inform the main trial design and, after recruitment rates were adjusted (calculated as 60% eligible, 20% of eligible patients recruited in the first 18 months, 45% thereafter for new centres), the main trial was given the green light to proceed.^25^ Further details of the progression criteria outlined in the By-Band-Sleeve study (and all examples of RCTs with an internal pilot discussed in this paper) are given in table 1.

Setting targets for recruitment-based progression criteria can be difficult and practice varies. Targets may be informed by pre-existing observational data on the number of incident cases expected during a defined period of time (eg, from national audit data or data collected by each centre) or on estimates from practising clinicians. An example is the NIHR-funded TARVA trial, which was designed to compare the clinical and cost-effectiveness of total ankle replacement versus ankle arthrodesis in patients with osteoarthritis.^23^ The progression criteria were informed by locally available data from centres on the number of eligible patients presenting annually. This was used to inform the minimum recruitment thresholds required to be achieved during the main trial.

A key decision relating to recruitment-based progression criteria is the proposed length of the internal pilot and duration of the recruitment period, the latter of which has been observed to range from 5 months^24^ to over 2 years.^16^ Often, however, the total period of the pilot phase is not reported. The duration may be influenced by the volume of patients and the nature (eg, adherence, variability of outcomes, combined event rates) of critical information needed. The time taken to open centres may also impact on timescales, and is influenced by the experience of the participating centres, ensuring that contracts are in place and the teams are able to deliver the interventions. A progression criterion may therefore include the number of centres that are expected to open within a specified period. A pilot phase needs to be sufficiently long to accommodate delays with opening centres and allow variations in patient flow to be documented over time to provide a fuller picture of the activity spread across a number of centres that would mimic the main trial. Consideration of whether recruitment rates have seasonal fluctuations is also recommended.

The issues discussed above are influenced by the number and type of centres that are expected to open and begin recruitment in the pilot within a defined period.^24^ Pilot centres may be chosen because this is where the co-applicants work, are close in proximity or they are where natural collaborations already in place.^17^ Selecting ‘home’ or otherwise safe and ‘enthusiastic’ centres brings potential benefits, including keeping more of the grant funding in house, thus ironing out difficult issues (eg, with contracts) with familiar staff before rolling out to other centres, and being able to visit centres promptly should issues arise. This may, however, lead to overestimation of recruitment that might not be replicated across centres participating in the main trial. Aiming for centres with a mixture of previous experience of recruitment success, enthusiasm or resources is recommended to reflect the reality of data collection. The ongoing NIHR-funded VIOLET study, which is comparing video-assisted thoracoscopic lobectomy and conventional open lobectomy for lung cancer,^24^ is an example of a surgical trial with an internal pilot in which the five pilot centres have been deliberately selected to include a range of research experience, to gain a realistic picture of feasibility.

#### Protocol non-adherence

A second set of progression criteria focus on the assessment of protocol non-adherence. Assessment may include all protocol procedures,^32^ although most often the focus is on adherence to the intervention. There are two main types of non-adherence: ‘cross-over’ and ‘off-protocol intervention’.^1^ Cross-over refers to a participant not receiving their allocated intervention and instead receiving an alternative trial intervention to which they were not allocated. Cross-over has the potential to reduce the power of the study because study groups may become more similar and the difference between overlapping groups is smaller than expected.^33^ ^34^ This may be overcome by inflating the sample size to account for a certain rate of cross-over,^34^ ^35^ so a progression criterion may relate to whether cross-over is within the rate allowed for in the sample size inflation. Off-protocol intervention describes the situation when another intervention (whether experimental or standard, a switch to another first-line intervention due to side effects from their allocated intervention or a second-line intervention due to disease progression) not included in the intervention protocol is used. Switches to off-protocol interventions may make the randomised groups more similar or more different in outcome.^36^ Non-adherence to the interventions may arise when patients or clinicians decline or cease the allocated intervention after randomisation and decide to select an intervention other than that allocated by randomisation (either in another trial group or an off-protocol intervention). In a trial in which patients are blinded to their allocation and/or the interventions on offer, it is less common for cross-over to occur, although a patient could request an intervention outside the study (off-protocol intervention). Non-adherence may also arise if there are problems with the acceptability of the intervention protocol, such as when side effects or adverse events are experienced.

Experience in the pilot phase may inform the main trial procedures to enhance adherence. It may also allow a fuller understanding of potential issues with the feasibility of the protocol and the overall trial design. An example is the ‘SUN-D’ trial (supported by the Grant-in-Aid by the Ministry of Health, Labour and Welfare, Japan), which investigated the optimal first-line and second-line antidepressant interventions for major depression.^15^ After the first 8 months of the multicentre pilot phase, investigators found that a proportion of patients had not reached their intended dosage by the scheduled time points. Minor changes were made to the main trial protocol to allow greater flexibility in the dose titration schedules and the internal pilot has since progressed to the main trial. The introduction of protocol modifications shows that knowledge of problems with intervention adherence can facilitate a successful main trial, though a further pilot period may be needed to show that the progression criteria are being met following a modification.

While the rate and direction of cross-over are often well reported in trials, details about off-protocol intervention are less so. This may be due to difficulties in obtaining this information or pragmatic decisions on what information to publish due to journal word count restrictions. Furthermore, formal quantifiable progression criteria set around maximum targets for cross-over or off-protocol intervention specifically (as opposed to the broader issue of non-adherence) are relatively rare.^14^ ^18^ ^26^ ^27^ In the NIHR-funded KORAL study, a placebo-controlled trial of arthroscopic lavage for osteoarthritis of the knee, rates of cross-over and withdrawal were monitored and contributed (along with other issues that emerged during the internal pilot, including a decline in the use of arthroscopic lavage in clinical practice) to the main trial being stopped, but formal quantifiable progression criteria set around cross-over were not detailed.^14^ Conversely, the ongoing NIHR-funded NERVES trial, a pragmatic trial with an internal pilot phase comparing nerve root injection with surgery for acute sciatica,^20^ did specify stop/go rules. They agreed in advance that if more than 50% of patients in the injection group cross-over to surgery, the TSC/Independent Data Monitoring Committee (DMC) will evaluate the reasons for cross-over and consider stopping the main trial. Recruitment-based progression criteria in NERVES were informed by Hospital Episode Statistics data for the numbers of surgeries performed annually in each centre.

Observed cross-over needs to be within that allowed for in the sample size calculation. However, uncertainty remains around how protocol adherence may be monitored and when non-adherence should result in modifications or prevent continuation to the main trial. These issues need to be considered on a trial-by-trial basis. Detailing the reasons for cross-over and off-protocol interventions received during an internal pilot will help inform the design of the main trial, for example, by alluding to problems with patient information, the feasibility of the intervention or assessment protocol, site training and recruitment processes. If non-adherence is a substantial issue, potentially compromising the statistical power of the main trial, it may be better to not progress to the main trial and reconsider its design (the issue of when to opt for other types of pilot studies is discussed below).

#### Outcome data

Monitoring the completeness and quality of short-term outcome data during an internal pilot provides a valuable opportunity to identify any problems and initiate changes to conduct to improve data collection in the main trial. Ideally, each trial participant will contribute complete data, collected at predetermined time points, as outlined in the protocol. In practice, however, missing or inadequate data are inevitable, meaning that some participants will not be included in the outcome assessment. The amount of missing data can have important implications for the trial's required sample size. The most common reason for missing or inadequate data is attrition or loss to follow-up (for reasons such as death, poor health, loss of interest, moving out of the area) or problems with the feasibility or acceptability of the outcome assessment protocol (eg, organisational issues, amount/timing of assessments) or the outcome measure instruments themselves (eg, lack of applicability, problems with comprehension). Even when some data are available, its quality may be poor; for example, items on a patient-reported outcome measure may be only partly completed or data may not have been collected promptly, resulting in only a small percentage of patients providing usable assessments within an acceptable time frame. Assessing rates of completeness of outcome data to report with the progression criteria can therefore be useful and may form a focal point for progression criteria in particular clinical settings.

For example, in the Putting Life in Years (PLINY) trial, funded by the NIHR, there was a prespecified target of collecting valid primary outcome data for 80% of those recruited at the 6-month follow-up in the internal pilot phase.^21^ Another example is the Sheffield trial of physical activity ‘booster’ interventions to sustain increased physical activity in middle-aged adults in deprived urban neighbourhoods. The progression criteria specified that at least 75% should provide 3-month follow-up measures, including the primary outcome measure, to ensure that the trial would be powered to show an effect if one existed.^22^ Despite not fulfilling this (and other) progression criteria, the main trial proceeded with design modifications (although the trial eventually closed early due to poor recruitment and retention of participants with primary outcome accelerometer data). This is potentially an example of an ‘amber’ situation following the internal pilot, where there may have been some reservations about proceeding to the main trial. In amber situations, there may be remediable issues that would otherwise prevent progression to the main trial but that, if identified early enough, can be addressed to the satisfaction of those reviewing progress in order for the trial to continue to a main trial.

In contrast, the COMMANDO trial,^19^ funded by the NIHR, was an example of an RCT that was stopped after the internal pilot. COMMANDO looked at changing eating behaviours in obese children, using a portable computerised weighing scale called a Mandolean that provided feedback to participants to promote normal patterns of eating and satiety. Progression criteria included that 36 or more families would be eligible for the main study, that 90% of patients randomised to Mandolean therapy would eat off the device at least five times a week, that using the Mandolean would reduce the speed of meal consumption within 3 months and that 80% of participants would provide data at their 3-month follow-up appointment. However, the pilot failed to meet any of its progression criteria and the trial was stopped.

In many cases, progression criteria set around key outcome data do not detail specific targets for completeness or quality^14–17^ ^22^ ^26–28^ ^37^ ^38^ or focus only on certain aspects of attrition.^15^ ^18^ ^21^ ^22^ Assessing the completeness and quality of outcome data in an internal pilot may not be appropriate if the primary outcome cannot be assessed in any or the majority of the patients within the lifespan of the internal pilot. Assessing shorter term follow-up may offer some insight into likely retention rates in the main trial but there is the potential for overestimating target retention rates.

### Approaches to the development and review of progression criteria

Current practice in the NIHR Health Technology Assessment (HTA) programme, which may be similar to other funding bodies, is for progression criteria to be preagreed between the trial team and funder at the application stage. This agreement will include a decision about the length of the internal pilot and when to submit data on progression criteria that can be discussed at a ‘monitoring visit’ such as those carried out by the NIHR HTA programme. It is important to carefully schedule (though with flexibility, if required) discussions about progression criteria to ensure that they are soon enough to avoid unnecessary effort and expenditure on an unfeasible study but that sufficient time has passed to gain useful information and to allow a reasonable time window for discussions and modification of the main trial or the progression criteria (or both) if required. While it is often easier to judge whether an internal pilot should proceed to a main trial, in practice whether an internal pilot has failed to achieve its target(s) requires a more complex judgement. Trials may fall into the amend zone due to a factor that severely reduces recruitment but is temporary and remediable (such as a trial manager having sickness absence) and therefore does not require stopping the trial. It is unlikely that a trial would be stopped according to a predetermined rule without considering whether there are remediable factors. In contrast, if a trial is clearly failing with no obvious remediable cause, then a decision to stop the trial may be reached more quickly.

Information or data relating to progression criteria should not be considered in isolation. Supplementary data may serve to facilitate decision-making about whether the main trial should proceed even when progression criteria have not been met. Funding bodies have acknowledged the value of considering not just whether studies have achieved their progression criteria but all relevant information on trial progress. Supplementary data that may be included in an internal pilot report may encompass how the trial team has responded to and overcome problems that have arisen (eg, overcoming unexpected excess intervention costs or barriers to recruitment). Funders may offer the opportunity to submit a ‘rescue plan’ that outlines how problems may be overcome for the main trial, with revised progression criteria.^39^ The protocol for the NIHR HTA-funded TARVA (total ankle replacement vs ankle arthrodesis for osteoarthritis) trial, for example, specifies that failure to meet the recruitment target set in the feasibility phase (ie, 10% of the recruitment target has not been reached 6 months into recruitment) will trigger submission of a plan to the funder, based on an options appraisal undertaken by the Trial Management Group and outlining the actions to be taken to improve recruitment to ensure that the target is met.^23^ Consideration would then be given to halting the trial had targets still not been met 15 months into recruitment. Alternatively, meeting all prespecified progression criteria at the end of the internal pilot phase does not automatically guarantee continuation to and throughout the main trial, as important factors (eg, progress with recruitment) will typically continue to be monitored throughout the entirety of the trial.

### The key role of the TSC

It may be argued that consideration of trial progression is not new and that this is routinely considered by a TSC. The TSC provides independent oversight of a trial on behalf of the sponsor and funder and considers and acts on the recommendations of the DMC, or equivalent. Rather than review progression criteria, however, the DMC considers information collated about the ongoing trial, including statistical criteria (often called stopping rules), and may recommend whether to stop the trial, typically on grounds of safety or efficacy, and sometimes futility,^40–42^ though often stopping rules do not consider futility or deliverability of the trial at such an early stage as the review of progression criteria in an internal pilot. The decisions and recommendations made by a TSC across a trial's lifespan may be considered to overlap somewhat with the formal review of progression criteria undertaken between the TSC, trial team and funder at the end of the internal pilot phase, yet there are important differences. The main difference is that the trial team has preagreed that if progression criteria are not being met, then the trial may be stopped. The added value of a trial with an internal pilot phase is, therefore, focus on achieving the criteria and, where problems arise, finding solutions earlier than if the trial was being conducted without an internal pilot. The TSC typically plays an integral role in how information about progression criteria is fed back to the funder, particularly if the trial is in the amber zone where progression to the main trial is uncertain. If this happens, ongoing communication between the trial team, the TSC and the funder may be necessary until it is agreed that amber issues have been resolved and it is considered appropriate that the main trial continues.

### Using changing evidence during the internal pilot phase of an RCT

The design of an RCT with an internal pilot phase will be based on current best evidence available during the planning stages. However, critically important new information may become available during the course of a trial that may be considered sufficient to require changes to its trial design. The use of an internal pilot design may serve as an added opportunity to make operational modifications to the main trial, for example, inclusion of a new intervention established during the internal pilot phase of a trial, where there is knowledge of the innovation of a new surgical technique. This is different from an adaptive trial design, which allows modifications to the design of the trial, its statistical procedures or its hypotheses that are typically planned in advance and possibly substantive.^42^

At the time the original By-Band study was being designed,^18^ interest in a novel type of bariatric surgery—laparoscopic sleeve gastrectomy—was growing. In response to this, progression criteria agreed in the internal pilot phase included a review of current practice of sleeve gastrectomy by the end of the pilot phase, to understand whether uptake and standardisation of the procedure was sufficient to extend the main trial to include a third intervention group and review of new evidence that may have accumulated in this area. The review showed a significant increase in the number of sleeve gastrectomy procedures being undertaken in the UK and, after the internal pilot phase, By-Band was adapted to a three group trial, By-Band-Sleeve.

### Reporting of internal pilot trials

The findings from pilot work, including RCTs with an internal pilot phase, are often reported as inconclusive and difficult to interpret.^10^ In addition, the assumption that pilot studies are less rigorous than main studies and are not designed to test the main trial hypothesis means that their findings are underdisseminated, thereby preventing triallists from accessing information that may be valuable to the success of future main trials.^3^ ^10^ The systematic reporting and publication of the design, results and evaluation of internal pilot trials in the literature should be encouraged to enable triallists to gain experience in interpreting the results of internal pilots and to learn from the experiences of internal pilots that have not progressed to a main trial (eg, where an RCT with an internal pilot is stopped early). These ‘failed’ trials may provide valuable insights into the types of challenges that triallists face when designing and conducting internal pilot trials that may inform future trials, in addition to saving resources on a main trial that is unlikely to succeed.^9^ ^14^ ^43^ ‘Pilot and feasibility studies’ is a new open access journal that is committed to ensuring that the results of all well-conducted, peer-reviewed, pilot and feasibility studies are published, regardless of the outcome or significance of the findings.^44^ Where an internal pilot has progressed to a main trial, the subsequent report of the main trial should clearly describe the internal pilot phase, so that its impact on the design of the main trial can be understood. Greater transparency in reporting how decisions were made to proceed with, amend or abandon the main trial, particularly in relation to the progression criteria, is also required. Recently, a new reporting guideline for external pilot and feasibility studies has been published as an extension to the CONSORT statement.^45^ ^46^ Similar guidelines for internal pilot studies may be useful to facilitate understanding of their purpose, enable journals to better evaluate the rigour of such studies and aid their interpretation in the literature.

Details provided in study protocols of the internal pilot phase of an RCT and any associated progression criteria also vary widely. Often, detail included in a funding application may not be included in the protocol or subsequent reports. It is also possible that progression criteria are only agreed with the funder after funding has been granted and the protocol developed, and so not included in the protocol. This raises issues about the level of detail that should be required in study protocols.

### Ten top tips for developing and using progression criteria for internal pilot studies

Ten top tips for developing and using progression criteria for internal pilot studies are proposed (box 1). These tips include suggestions for specifying and assessing progression criteria and the detailed reporting of internal pilot studies.

Box 1 **Ten top tips for developing and using progression criteria for internal pilot studies**

1. A traffic light system of green (go), amber (amend) and red (stop) might be preferable to a simple stop/go approach when specifying progression criteria for internal pilot studies;

2. Pre-specified progression criteria agreed with funders need to strike a careful balance between being firm enough to promote ambition in the trial team yet being flexible enough to allow opportunities to remedy early problems;

3. Recruitment progression criteria should be based on rates per centre per unit time (eg, per month) that can be easily extrapolated, rather than specifying that an absolute number should be reached by a specific date, due to the unpredictability of opening sites;

4. When recruitment falls behind, it is essential to explore screening logs to determine whether insufficient participants were approached, insufficient participants passed eligibility criteria or insufficient eligible participants agreed to randomisation;

5. The assessment of intervention adherence, cross-over and outcome event rates should take into account the duration from randomisation to timing of primary outcomes if sufficient data are to be gleaned in time to inform progression;

6. When assessing missing data, it is important to explore the degree of missing data within key outcomes as well as the percentage of participants with missing data;

7. Trial teams should involve both their funders and their Trial Steering Committee in assessing their progression criteria;

8. Pilot study recruitment sites should be representative of sites that recruit into the main study;

9. Triallists may be able to take the opportunity to assess whether changes to existing technologies have occurred since the original study was planned, so that new technologies can be considered with funders, such as using an adaptive design;

10. Pilot studies need to be reported fully. An extension to CONSORT guidelines for pilot and feasibility studies is now available.

## Discussion

An internal pilot phase within an RCT allows key aspects of trial conduct to be established in a subset of centres before expansion into the main trial. The preagreement of stop/amend/go progression criteria to be reported to the funder provides a focus to optimise pilot work and the subsequent main trial. A formal internal pilot trial design also allows a trial to be abandoned if key criteria demonstrate that a main trial is not realistic to undertake even with some modifications in design. Progression criteria predominantly focus on trial recruitment, although protocol adherence and follow-up criteria are often assessed and reported. This paper has summarised the key issues to consider in the development of operational progression criteria for internal pilot studies, in order to inform efficient RCT design. Previously, guidance in this area has been lacking, accompanied by uncertainty about how progression criteria should be applied and reviewed. Top tips for developing and using progression criteria for internal pilot studies are proposed.

### When to use an internal pilot

Designing an RCT with an internal pilot phase is unlikely to be appropriate in most situations where *substantive* changes to key components of the trial, such as the intervention or outcomes, are likely to be required but is appropriate where there is some uncertainty around key areas that could influence the success of the trial, such as recruitment. They may also be recommended where participating centres and chief investigators have limited experience with trials. While data collected during an internal pilot may inform decisions to make minor modifications to the design and conduct of the main trial phase (eg, to sampling and/or recruitment or the intervention protocol), such modifications should not be expected to reduce the ability of the pilot data to contribute to the main study.

An internal pilot may also be considered where teams have limited experience of conducting multicentre trials. The pilot phase then also acts as an opportunity to test whether successful collaborations can be developed and trial processes run across multiple centres. This has been exemplified recently with surgical teams in the UK beginning to collaborate, design and conduct RCTs when previously it was uncommon. Over the past decade, there has been a steady increase in the number of NIHR multicentre trials funded and many are designed with an internal pilot phase.

Unexpected problems resulting in substantive changes to recruitment, protocol adherence, outcome data or other aspects of design, particularly the intervention and the outcomes, may prevent inclusion of data from the internal pilot in the main trial analyses. It is therefore important to know in advance of the internal pilot phase which outcomes will be measured in the main trial and how, so that relevant data are collected throughout. When there is considerable uncertainty or risk around the design of the main trial, or when substantive changes are anticipated, other types of pilot work that allow for more substantial design amendments, such as an external pilot trial, are likely to be more appropriate. For example, where uncertainties about the availability of eligible patients or the ability to recruit prevent producing reliable recruitment targets, the NIHR HTA recommends that researchers consider applying for an external rather than an internal pilot.^39^ However, there is a place for more pragmatic trials, which allow greater variation in trial procedures in order to maximise generalisability and provide evidence relevant to clinical practice and policy if transparently designed and reported.^47^ Recent work to develop a checklist for systematically documenting design modifications during pilot trials may help to inform decisions about whether it is advisable to pool data from the pilot phase and the main trial.^47^ Further work to categorise the magnitude of design modifications to an RCT with an internal pilot phase and to develop recommendations to facilitate decision-making about pooling data is ongoing.^47^ Work to further refine definitions of different types of pilot and feasibility studies is needed to support decision-making regarding the design of pilot work for RCTs.

While developed with RCTs with an internal pilot phase in mind, it is possible that the recommendations for progression criteria presented in this paper may also be applicable to decisions regarding the main trial conduct and design following an external pilot study.

### Practicality of RCTs with an internal pilot phase

When designing a trial with an internal pilot phase, triallists should take care to ensure that the use of progression criteria, which will often quantify targets (eg, for the recruitment of patients or opening of centres), does not counteract progress. Often, an internal pilot phase will be conducted in a subset of all centres to be opened within the main trial phase, and a progression criterion may include the number of centres to be opened within a given period. If, however, it is possible to open additional centres during that time, then it may be detrimental to the trial not to do this. Consideration should also be given to ensure that the time taken to prepare the report from the internal pilot phase and for the trial team, TSC and funder to review progress against progression criteria does not inhibit seamless transition to the main phase of the trial.

### Better reporting of internal pilot RCTs is needed

Currently, detailed reporting of progression criteria, and how funders review and act on information about progress against these criteria, is uncommon and it is difficult for triallists to learn from past experience. It is recommended that progression criteria are reported clearly in a trial protocol paper and subsequent protocol update papers and trial reports, alongside how trial teams met/did not meet criteria and how the funding body responded to this information, to inform the future development, presentation and optimisation of criteria in trial design.

## Conclusions

Designing studies with an internal pilot phase may optimise the use of pilot work to inform more efficient RCTs. The careful selection of preagreed progression criteria provides a valuable opportunity to formally review complex trial processes, inform decisions about trial conduct and whether continuation to the main trial is appropriate. This has the potential for the trial team and funder to identify and address problems that may jeopardise the success of the main trial sooner than would have been possible without an internal pilot phase. It has the added potential to avoid waste of valuable resources. This paper outlines common issues to consider in the optimal development and review of operational progression criteria. Describing the internal pilot phase of an RCT in trial protocols is recommended, and detailed and systematic reporting of internal pilot studies is encouraged to aid triallists in their design, and to facilitate their evaluation by journals and their interpretation in the literature. Transparent reporting around the choice of trial design and the decision-making process for stopping, amending or proceeding to a main trial should be regarded by the research community as beneficial to facilitating understanding of the appropriate and optimal use of RCTs with an internal pilot phase.
